# The effect of psyllium consumption on blood pressure: Systematic review and dose–response meta‐analysis of randomized controlled trials

**DOI:** 10.1002/fsn3.3863

**Published:** 2024-08-29

**Authors:** Zeinab Gholami, Zamzam Paknahad

**Affiliations:** ^1^ School of Nutrition and Food Sciences, Students' Research Committee Isfahan University of Medical Sciences Isfahan Iran; ^2^ Department of Clinical Nutrition, School of Nutrition and Food Sciences Isfahan University of Medical Sciences Isfahan Iran

**Keywords:** blood pressure, diastolic blood pressure, psyllium, systolic blood pressure

## Abstract

Based on available evidence, psyllium has been found to play a role in preventing and improving hypertension. In light of this, the objective of this investigation is to perform a systematic review and meta‐analysis to assess the effect of psyllium intake on blood pressure. In order to identify suitable publications, we conduct searches using Scopus, ISI Web of Science (WOS), and PubMed, and from 15 March 2022 to 15 July 2022. This study aims to evaluate the impact of psyllium consumption on blood pressure in adults through randomized controlled trials (RCTs). We used the fixed‐effects model which are expressed as weighted mean differences (WMD) with 95% confidence intervals (CI). In this article, 14 RCT studies and 802 participants were included. Psyllium consumption significantly decreases systolic blood pressure (SBP): (weighted mean difference [WMD]: −2.24; 95% CI: −3.13, −1.35; *p* < .05), and non‐significantly increases diastolic blood pressure (DBP): (WMD: 0.04; 95% CI: −0.52, 0.61; *p* > .05). Psyllium dosage and duration of consumption had a remarkable linear effect on SBP and DBP. Results showed a significant decrease in SBP and a non‐significant increase in DBP following psyllium consumption.

## Graphical abstract

Psyllium consumption significantly decreases systolic blood pressure (SBP) and non‐significantly increases diastolic blood pressure (DBP).

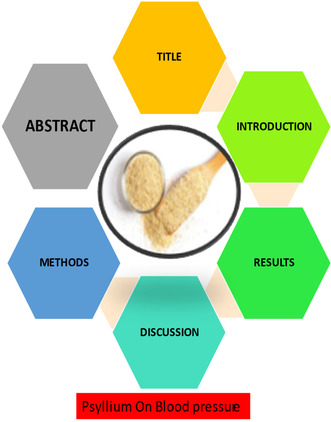

## 1 INTRODUCTION

Hypertension poses a significant public health challenge in both economically developing and developed countries. A considerable proportion of hypertensive individuals remain undiagnosed, while those with diagnosed hypertension are also affected (Kearney et al., 2004). Hypertension is a common chronic age‐related disease that is often associated with severe cardiovascular and renal complications. Studies indicate that systolic blood pressure exhibits an age‐related increase until the eighth decade of life. However, diastolic blood pressure experiences a rise only up to 50 years of age, following which it either stabilizes or shows a slight decrease (Staessen et al., 2003). The global incidence of hypertension demonstrates variability, with rural India exhibiting the least prevalence and Poland the highest (Kearney et al., 2004).

For dietary fiber, it has been argued that the incorporation of both dietary fiber and functional fiber is a crucial factor (Gibb et al., 2015).

The overall effect of viscous soluble fiber is lower SBP and DBP. By improving blood pressure, adding viscous fiber to regular diets may also help lower the risk of cardiovascular diseases (Khan et al., 2018). The psyllium plant is once again being grown and sold commercially in the Indian subcontinent. Because of their small size and resemblance to a horse's ear, this plant's seeds have been known throughout as “horse's ears” or “Isabgul”. Psyllium has new applications today in addition to its more well‐known ones, including those in industry, medicine, and even some global regions where it is grown. It has a very broad and specialized following (Naghdi Badi et al., 2004). Psyllium constitutes a dietary fiber that is soluble and mucilaginous in nature (Singh, 2007)—the substance in question is a mucilage that forms a gel and is extracted from the seed husk of *Plantago ovata* (Belorio & Gómez, 2021; Jovanovski et al., 2018; Olson et al., 1997). Psyllium is a naturally occurring isolated fiber that is primarily soluble and gels when moistened. The psyllium gel does not ferment and makes it through the entire digestive system undamaged. The psyllium gel makes chyme viscous in the small intestine, which slows nutrient breakdown and absorption (McRorie Jr et al., 2021). The ground skin of psyllium seeds consists of a combination of polysaccharides such as hexoses, pentoses, and uronic acids. This has been utilized as a non‐fermented fiber supplement that can form a viscous, soluble gel (Darooghegi Mofrad et al., 2020). Concentrated fibers are derived naturally from psyllium. The mechanisms of the effects of psyllium are comparable to those of other fibers talked about, along with a rise in bile acid excretion (stimulating 7‐hydroxylase) and a decrease in absorption of cholesterol in the intestine (Cicero et al., 2021). Psyllium helps hypertensive patients lower their blood pressure. (Gibb et al., 2023). Psyllium is typically found growing in its natural habitat in India, Iran, and other countries located in the Middle East (Tabrizi et al., 2005; Wei et al., 2009). Psyllium has benefits such as treatment of diarrhea, constipation, inflammatory bowel disease, hypercholesterolemia, and diabetes. Studies showed that psyllium consumption decreases blood glucose levels after one dose consumption (Belorio & Gómez, 2021; Darooghegi Mofrad et al., 2020; Gibb et al., 2015; Singh, 2007). Consumption of both soluble and insoluble fibers leads to decrease in blood pressure (Lupton & Turner, 2003). Consuming psyllium reduces the risk of developing many cardiovascular diseases, including high cholesterol, hypertriglyceridemia, hyperglycemia, and hypertension (Cicero et al., 2007). In the treatment of cardiometabolic diseases like hyperlipidemia, diabetes mellitus, and its complications such as hypertension, hyperuricemia, and obesity, as well as in the use of food systems, psyllium is used (Chen et al., 2022). In high‐normal blood pressure subjects who consume a lot of salt, regular consumption of dietary fiber made from psyllium husk lowers SBP (Yoshinuma et al., 2019). The addition of fiber to one's diet may yield favorable outcomes on vascular health and blood pressure. A study was conducted to investigate the impacts of 12‐week psyllium fiber consumption on blood pressure and vascular function in obese and overweight persons. However, the results of this study did not indicate any significant improvements in either of these markers (Pal et al., 2012). The consumption of psyllium supplementation led to a notable reduction in SBP, while there was no significant decrease observed in DBP (Clark et al., 2020). But in another study, 6 months of administration of psyllium fiber showed a significant decrease in both SBP and DBP among overweight individuals with hypertension (Cicero et al., 2007).

Due to the contradictory nature and limited quantity of research in this area, a systematic review and meta‐analysis study are deemed necessary to obtain a comprehensive understanding. Therefore, the aim of our study is to investigate the impact of psyllium consumption on blood pressure in adults through a systematic review and dose–response meta‐analysis.

## 2 METHOD

### 2.1 Search strategy and study selection

The research adhered to the rules and regulations established by the Preferred Reporting Items for Systematic Reviews and Meta‐Analyses (PRISMA) guideline (Parums, 2021). In order to evaluate the impact of psyllium on changes in SBP and DBP, a search for relevant studies was conducted across four English language databases (Web of Science (WOS), Scopus, and PubMed) from their inception until July 15th, 2022. The data were meticulously obtained by utilizing specific keywords: (“Psyllium” OR “Plant Mucilage” OR “mucilage” OR “lunelax” OR “Metamucil” OR “ispaghul” OR “plantago” OR “isogel” OR “ispaghula” OR “psyllium‐husk” OR “Plantago ovata” OR “Psyllium fiber” OR “Plantago psyllium” OR “mucilage polysaccharides”) AND (“Randomized Controlled Trial” OR “Clinical Trial” OR “cluster randomized controlled trials” OR “RCTs” OR “cRCTs” OR “Controlled Clinical Trial” OR “RCT” OR “double‐blind randomized controlled trial” OR “Clinical Trials as Topic” OR “clinical trial*” OR “controlled trial*” OR “intervention*” OR “Randomized” OR “Randomized” OR “randomly” OR “single‐blind” OR “double‐blind” OR “placebo” OR “Pilot study” OR “single‐blind randomized controlled trial” OR “Controlled Clinical Trials as Topic” OR “Meta‐Analysis” OR “Review” OR “Random Allocation” OR “Single‐Blind Method” OR “Double‐Blind Method” OR “Cross‐Over Studies” OR “Comparative Study” OR “Follow‐Up Studies” OR “cross‐over” OR “parallel” OR “assignment” OR “trial”) alone or combined together with “OR” and/or “AND”. In order to locate supplementary studies, we examined the reference lists of the articles that were retrieved. To ensure precision, our search was limited to studies involving human subjects exclusively. In order to avoid duplications, two independent researchers (Z.Gh. and Z.P.) reviewed both primary titles and abstracts. Moreover, we conducted a manual search for supplementary articles in the gray literature and utilized the alert system of Scopus and PubMed databases. Corresponding author(s) were contacted via email for articles that were not accessible to us.

### 2.2 Eligibility criteria

This meta‐analysis study employed the population, intervention, comparison, outcomes, and study (PICOS) criteria. Accordingly, the study design (randomized controlled trials [RCTs]) included adults who were over 18 years old, intervention (psyllium), comparison (placebo), and outcome (alteration in SBP and DBP levels). The following criteria were assessed: (a) adults who were ≥18 years; (b) evaluated the effect of psyllium on SBP and DBP changes with a control group; and (c) RCTs with either parallel or crossover design. Exclusion criteria were as follows: (a) persons who were less than 18 years old; (b) cell culture, animal, or in vitro studies; (c) articles that were not RCT; (d) studies that were seminars, conferences, letters, reviews, and abstracts with defective data; (e) defective data; (f) articles without expression standard deviation (SD); (g) articles without control group; (h) articles whose study duration is less than 2 weeks; (i) articles that were not in English; and (j) articles that had no baseline mean and SD.

### 2.3 Data extraction

Two review authors (Z.Gh. and Z.P.) completed the data extraction form using both a word processor and spreadsheet. The authors conducted a thorough review of the selected papers. An email was sent to the corresponding author(s) to acquire full‐text articles that were inaccessible. After successfully conducting a review of the full text, the information including design of the study (parallel or cross‐over), study location, the publication year, author's name, the population of study, health status of participants, mean age of the participants, sample size, gender, the mean ± SD of the SBP and DBP levels before and after the intervention, duration of intervention, and psyllium dosage were extracted. If there were studies that included an extra arm, they are documented as distinct reports, as indicated in Table 1. In cases where the numerical values for mean and standard deviation were not provided, data were extracted from published figures through the use of the Graph Digitizer software.

**TABLE 1:** General specifications of the recognized studies used in meta‐analysis.

Author	Year	Country	Population	Sex	Prevalence by sex	*N*	In_*N*	Pl_*N*	In_Mean age	Duration (day)	Type of psyllium	Type of pl	Dose/day (g/day)
Ong et al. (Kearney et al., 2004)	2022	Malaysia	Healthy male adult	M	100% M	29	14	15	26	30	Psyllium husk	Mixed herbs	25
Gary et al. (Staessen et al., 2003)	1988	Tennessee	Patient with cholesterol	M/F	27.7% M 72.3% F	54	27	27		49	Psyllium in diet		20.4
James et al. (Gibb et al., 2015)	1988	Lexington	Hypercholesterolemia	M	100% M	28	14	14	47.6	70	Psyllium hydrophilic mucilloid	Cellulose placebo	10.2
Arrigo et al. (Khan et al., 2018)	2007	Italy	Severe hyperlipoproteinemia; uncontrolled diabetes	M/F	50% M 50% F	96	48	48	48	180	Soluble psyllium husk powder	Hydrolyzed guar gum	7
Arrigo et al. (Naghdi Badi et al., 2004)	2010	Italy	Caucasian patients	M/F	50% M 50% F	96	48	48	48	180	Soluble psyllium husk powder	hydrolyzed guar gum	7
David et al. (Singh, 2007)	1997	Toronto	Healthy subject	M/F	46.87% M 53.13% F	32			57.5	28	Psyllium +6% mufa	Wheat bran	11.4
David et al. (Singh, 2007)	1997	Toronto	Healthy subject	M/F	44.44% M 55.56% F	27			58	28	Psyllium +12% mufa	Wheat bran	12.4
Sebely et al. (Belorio & Gómez, 2021)	2012	Australia	Overweight and obese	M/F	43.85% M 56.15% F	31	16	15	41.3	84	Psyllium fiber (FIB) supplementation	(starch)	21
Rosa et al. (Jovanovski et al., 2018)	2010	Spain	CVD	M/F	44.5% M 55.5% F	187	94	93	54.24	56	*Plantago ovata* husk	Microcrystalline‐cellulose	14
Sartore et al. (Olson et al., 1997)	2009	Italy	Type II diabetes	M/F	67.5% M 32.5% F	40	20	20	61	56	Psyllium	Diet alone	10.5
Damian et al. (McRorie Jr et al., 2021)	2021	Poland	Obese women	F	100% F	72	35	37	50.91	84	Plantago major	Microcrystalline cellulose	
Thomas et al. (Darooghegi Mofrad et al., 2020)	1994	Canada	Raised serum cholesterol	M/F	50% M 50% F	18				14	Psyllium fiber	Wheat bran	7.3
Larry et al. (Cicero et al., 2021)	1989	Ohio	Moderate hypercholesterolemia	M/F	50.66% M 49.34% F	75	40	35	46.2	56	Psyllium		10.2
Ricklefs et al. (Gibb et al., 2023)	2017	USA		M/F	52.94% M 47.06%F	17	8	9	58.5	56	Ground psyllium	Ground flaxseeds	9

Abbreviations: CVD, cardiovascular diseases; F, female; g, gram; In *N*, intervention number; IN, intervention; M, male; mufa, monounsaturated fatty acid; *N*, number; Pl *N*, placebo number; PL, placebo.

### 2.4 Quality assessment

The risk of bias in a study was assessed by utilizing the Cochrane collaboration's risk of bias assessment tool (Higgins et al., 2011). Seven criteria were assessed for each study that was taken into consideration: (a) allocation concealment, (b) random sequence generation, (c) blinding of outcomes assessment, (d) blinding of participants and personnel, (e) selective outcome reporting, (f) incomplete outcome data reporting, (g) general risk bias, and (h) other potential threats to validity. So, studies were ascribed as high quality (low risk of bias for all seven domains), moderate quality (unclear risk of bias for one or two domains), and low quality (low risk of bias for less than two domains) (Higgins et al., 2011) (Table 2).

**TABLE 2:** Quality assessment.

Article	Random sequence generation	Allocation concealment	Blinding participant and personal	Blinding of outcome assessment	Incomplete outcome data	Selective outcome reporting	Other potential threats to the validity	General risk bias
Wen et al. (2022)	L	H	H	H	L	L	H	H
Neal and Balm (1990)	H	H	H	H	L	L	H	H
Anderson et al. (1988)	H	H	L	H	L	L	L	H
Cicero et al. (2007)	L	H	L	H	L	L	L	M
Cicero et al. (2010)	L	H	L	H	L	L	L	M
Jenkins et al. (1997)	H	H	H	H	L	L	L	H
Jenkins et al. (1997)	H	H	H	H	L	L	L	H
Pal et al. (2012)	L	H	H	H	L	L	L	H
Solà et al. (2010)	L	H	L	H	L	L	L	M
Sartore et al. (2009)	L	H	H	H	L	L	H	H
Skrypnik et al. (2021)	L	L	L	H	L	L	H	M
Wolever et al. (1994)	L	H	H	H	L	L	L	H
Bell et al. (1989)	L	H	L	H	L	L	H	H
Ricklefs‐Johnson et al. (2017)	L	H	H	H	L	L	L	H

Abbreviations: BP, blood pressure; H, high; L, low; M, medium.

### 2.5 Data synthesis and statistical analysis

Our objective was to evaluate alterations in SBP and DBP values by means of mean changes and standard deviations (SD), employing a random‐effects model. (DerSimonian & Laird, 1986). In order to obtain precise measurements of pooled prevalence rates along with 95% confidence intervals, a random‐effects model was employed. The level of variation among the studies was examined through the use of comprehensive meta‐analysis (CMA) software. An *I* ^2^ value greater than 50% indicates a significant level of heterogeneity and suggests the application of a random‐effects model. To identify the sources of heterogeneity, we conducted separate meta‐regression and subgroup analyses. Specifically, meta‐regression was employed to investigate the impact of psyllium dosage and study duration. We assessed the publication bias with Begg's and Egger's tests. Therefore, there was no publication bias. A significance level of *p*‐value <.05 was applied to all statistical analyses, and the meta‐analysis was performed utilizing CMA version 3. In cases where the standard deviation of the mean difference was absent from the published literature, we applied a specified formula: SD change = square root ([SD baseline]^2^ + [SD final]^2^ – [2R × SD baseline × SD final]) (Borenstein et al., 2005). For calculating SD from SE, we used the following formula: SD = SE * $\sqrt{n}$. In instances where data are presented in graphical form without accompanying average and standard deviation information, the get data Graph digitizer software may be utilized to extract the necessary data. For considering heterogeneity, we used the *I* square (*I* ^2^) index. Accordingly, (*I* ^2^ > 75%), (*I* ^2^ = 50–75%), (*I* ^2^ = 25–50%), and (*I* ^2^ < 25%) were considered high, severe, moderate, and lowly heterogeneous, respectively (Higgins et al., 2003). We performed predefined subgroup analyses based on sample size, persons' mean age, study duration (days), psyllium dosage (mg/day), health status, the baseline SBP and DBP levels, sensitivity analysis, and publication bias.

## 3 RESULTS

### 3.1 Search results

**FIGURE 1:**
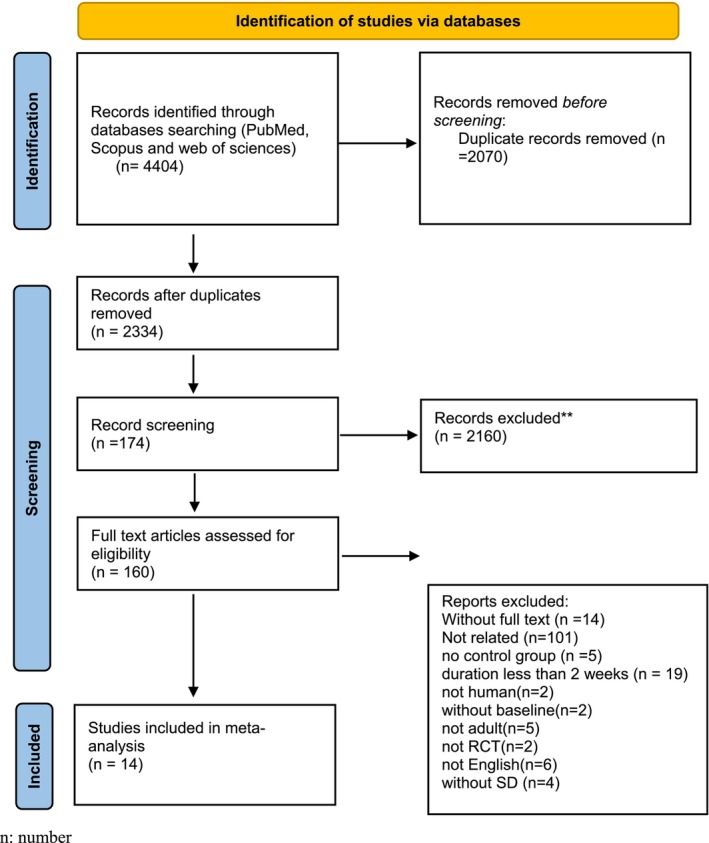
Flow diagram of study selection.

This study is registered in PROSPERO, under code CRD42023417492. It has received prior approval from student research committee, Isfahan university of medical sciences (Code: IR.MUI.RESEARCH.REC.1402.048, Grant number: 140222). The process for screening and selecting studies is presented in Figure 1 through a flowchart. To avoid missing any potentially relevant papers, the alert function in both PubMed and Scopus databases was activated. We removed 2334 duplicate articles (2070), and the titles and abstracts were subsequently scrutinized (2160). Next, 160 full‐text articles were screened. We excluded 141 studies, where 42 studies were not related, 5 studies did not have a control group, 2 studies worked on animals, 2 studies had no baseline mean and SD, 5 studies did not work on adults, 6 studies were not written in English, 2 studies were not RCTs, and 4 studies had no SD. Therefore, 14 RCTs were entered in the final meta‐analysis (Figure 1).

### 3.2 Study characteristics

The 14 eligible studies were published from 1988 to 2022 and were 14–180 days in duration. A total of 802 individuals participated in the study (consisting of 364 cases and 361 controls). Table 1 displays the general characteristics of these studies which were conducted in various countries: Malaysia, Lexington, Italy, USA, Spain, Tennessee, Toronto, Australia, Poland, Ohio, and Canada. The age range of participants varied between 26 and 61 years with a mean value. Most studies were conducted for both genders. The dosages of psyllium utilized in the included studies ranged from 7 to 25 g/day.

### 3.3 Meta‐analysis results

**FIGURE 2:**
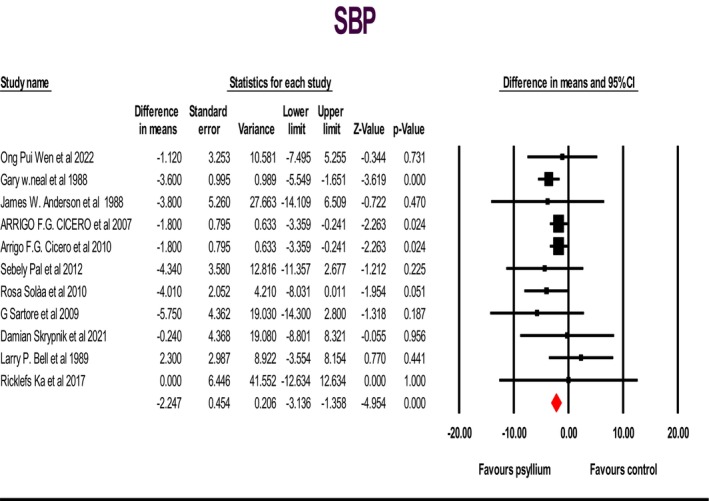
Forest plot illustrating weighted mean difference and 95% confidence intervals for the impact of psyllium consumption on systolic blood pressure.

**FIGURE 3:**
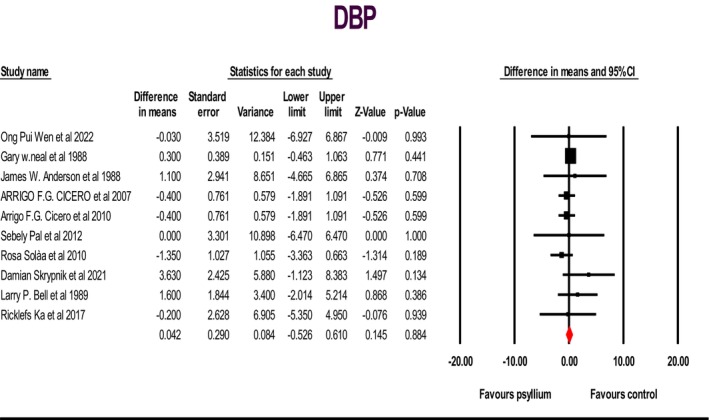
Forest plot illustrating weighted mean difference and 95% confidence intervals for the impact of psyllium consumption on diastolic blood pressure.

Fourteen studies were analyzed, comprising 802 individuals. These studies sought to investigate the impact of psyllium supplementation on alterations in systolic blood pressure (SBP) and diastolic blood pressure (DBP) levels. The fixed‐effects model was employed, which indicated a significant decrease in SBP: WMD: −2.24; 95% CI: −3.13, −1.35; *p* < .05 (Figure 2); and a non‐significant increase in DBP: WMD: 0.04; 95% CI: −0.52, 0.61; *p* > .05 (Figure 3). However, a non‐significant grade of low heterogeneity was discovered for SBP: (*I* ^2^ = 25.87%, *p* > .05) and DBP: (*I* ^2^ = 0.00%, *p* > .05). Sensitivity analysis showed that the sequential deletion of each study did impact SBP: WMD altered between −2.46 and −1.89 and DBP WMD altered between −0.27 and 0.11 (Figure S1).

### 3.4 Subgroup analysis

The studies were categorized according to the participants' BMI, study duration (days), baseline SBP and DBP levels (mean ± SD), and psyllium dosage (g/day). Subgroup analyses illustrated diversities in the effects of psyllium. SBP was significantly changed in subgroup with less and more than 10 g/day consumption (*p*‐value <.05) and showed significant change in subgroup with lower than and upper than 50 days consumption duration (*p*‐value <.05); DBP was non‐significantly changed in subgroup with less and more than 10 g/day consumption (*p*‐value >.05) and it did not show significant change at subgroup with lower than and upper than 50 days consumption duration *t* (*p*‐value >.05) (Figures S2 and S3). The subgroup analyses' outcomes are condensed in Table 3.

**TABLE 3:** Subgroup analysis.

Variable	Dose		Duration	
	<10 g/day	>10 g/day	<50 day	>50 day
SBP				
Number of comparisons	3	7	2	9
WMD (95% CI)	−1.78 (−2.88, −0.68)	−3.21 (−4.75, −1.67)	−3.88 (−5.25, −1.52)	−1.91 (−2.92, −0.90)
*p*‐Value	.00	.00	.00	.00
*I* squared	0.00	0.00	0.00	0.00
*p*‐heterogeneity	.96	.60	.46	.79
DBP				
Number of comparisons	3	6	2	8
WMD (95% CI)	−0.39 (−1.42, 0.64)	0.15 (−0.52, 0.84)	0.29 (−0.46, 1.05)	−0.28 (−1.14, 0.57)
*p*‐Value	.45	.65	.44	.51
*I* squared	0.00	0.00	0.00	0.00
*p*‐heterogeneity	.99	.69	.92	.66

Abbreviations: DBP, diastolic blood pressure; SBP, systolic blood pressure; WMD, weighted mean differences.

### 3.5 Dose–response analysis

**FIGURE 4:**
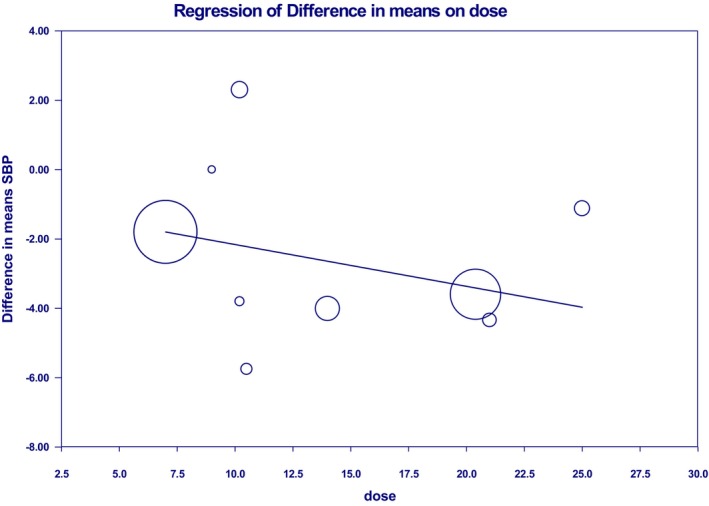
Dose–response relations between psyllium dosage (mg/day) and mean difference in systolic blood pressure.

**FIGURE 5:**
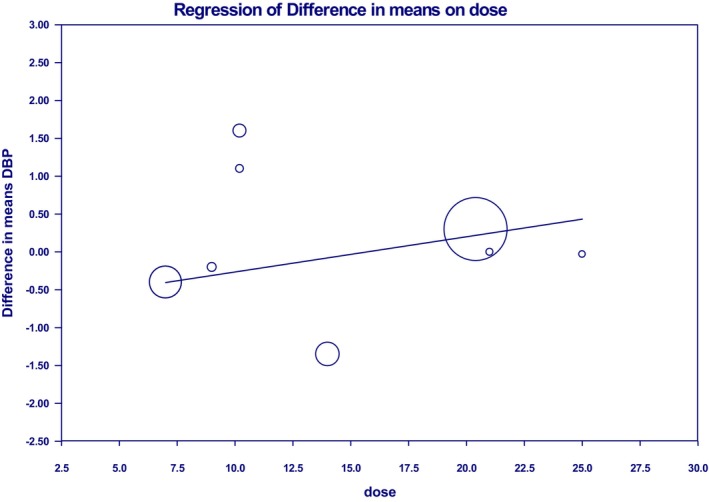
Dose–response relations between psyllium dosage (mg/day) and mean difference in diastolic blood pressure.

Psyllium dosage and duration of consumption had a remarkable linear effect on SBP and DBP; but not significant (Figures 4 and 5 and Figure S4). The consequences of the meta‐regression analyses, to the overall effect, are shown in (Table 4).

**TABLE 4:** Meta‐regression.

Variable	Slop	95% CI	*p*‐Value
SBP			
Dose	−0.12	−0.27, 0.03	.11
Duration	0.01	−0.004, 0.02	.16
DBP			
Dose	0.04	−0.04, 0.14	.33
Duration	−0.00	−0.01, 0.00	.28

Abbreviations: CI, confidence interval; DBP, diastolic blood pressure; SBP, systolic blood pressure.

### 3.6 Publication bias

The assessment of publication bias is illustrated in the plots. However, Egger's test disclosed no evidence of publication bias in studies examining the effect of psyllium consumption on SBP (*p* = .95) and DBP (*p* = .69). Begg's test disclosed no evidence of publication bias in studies examining the effect of psyllium consumption on SBP (*p* = .81) and DBP (*p* = .92) (Figure S5). Therefore, trim and fill analysis was performed; the results of this analysis with one imputed study showed that psyllium had more robust impact on decreasing DBP (WMD: −0.009, 95% CI: −0.93, 2.37) after considering publication bias. However, after trim and fill analysis, the SBP (no imputed study) was decreased. The results of the publication bias analysis, as well as the overall effect, are shown in Table 5.

**TABLE 5:** Publication bias.

Variable	Corrected effect size			Begg			Egger					Fail safe *n* test
	Study trimmed	WMD	95% CI	KENDALL TAU	*Z* value	*p*‐Value 2 tailed	Intercept	95% CI	*T* value	*df*	*p*‐Value	*n*
SBP	0	−2.24	−3.13, −1.35	−0.05	0.23	.81	−0.02	−0.99, 0.94	0.05	9.00	.95	33.00
DBP	1	−0.009	−0.57, 0.55	0.02	0.08	.92	0.17	−0.81, 1.15	0.40	8.00	.69	0.00

Abbreviations: CI, confidence interval; DBP, diastolic blood pressure; SBP, systolic blood pressure; WMD, weighted mean differences.

### 3.7 Quality assessment

Table 2 displays the outcomes of the quality assessment of the trials. Upon scrutinizing the quality of all trials that were incorporated, 10 were appraised as having low quality, while the remaining 4 studies were evaluated as possessing medium quality.

## 4 DISCUSSION

The systematic review and meta‐analysis revealed a significant reduction in SBP and an insignificant increase in DBP after psyllium ingestion compared to the placebo. There was a non‐significant low heterogeneity for SBP and DBP. The robustness of the findings was further demonstrated through sensitivity analysis. Specifically, the results indicated that the association remained unchanged despite the sequential removal of individual studies. This was particularly evident in relation to SBP (WMD altered between −2.46 and −1.89) and DBP (WMD altered between −0.27 and 0.11) as the sensitivity analysis displayed strong robustness.

The diastolic pulsatile increase in arterial blood pressure cannot be attributed to the contraction of the left ventricle, which is in a relaxed state and is disconnected from the aorta by the closed sigmoid valve. Also, as indicated above, it is not a reflection of the systolic pulse pressure wave, as it occurs earlier in the aorta than in the other arteries. Thus, the diastolic pulsatile increase in arterial blood pressure is the result of the sequential contraction of the arterial tree that occurs first in the aorta and later in the other arteries (Mandoki et al., 2013). However, numerous pathways probably contribute to the observed reduction in blood pressure, and further mechanistic studies are needed to elucidate the putative pathways and mechanism of action (Clark et al., 2020).

The studies were categorized according to the baseline SBP and DBP levels (mean ± SD), psyllium dosage (g/day), study duration (days), and participants' BMI. The statistical analyses conducted failed to identify any sources of heterogeneity. Psyllium dosage and duration of consumption had a remarkable linear effect on SBP and DBP, but it was not significant. Egger's test disclosed no evidence of publication bias in studies examining the effect of psyllium consumption on SBP and DBP. Begg's test disclosed no evidence of publication bias in studies examining the effect of psyllium consumption on SBP and DBP. Therefore, trim and fill analysis was performed—the results of this analysis with one imputed study showed that psyllium had more robust impact on decreasing DBP after considering publication bias. However, after trim and fill analysis, the SBP (no imputed study) was decreased.

Psyllium counteracts a large number of cardiovascular risk factors such as hypertriglyceridemia, high cholesterol, hypertension, and hyperglycemia (Cicero et al., 2007).

The overall effect of viscous soluble fiber is lower SBP and DBP. By improving blood pressure, adding viscous fiber to regular diets may also help lower the risk of cardiovascular diseases (Khan et al., 2018). Concentrated fibers are derived naturally from psyllium. The mechanisms of the effects of psyllium are comparable to those of other fibers talked about, along with a rise in bile acid excretion (stimulating 7‐hydroxylase) and a decrease in absorption of cholesterol in the intestine (Cicero et al., 2021). Psyllium helps hypertensive patients lower their blood pressure. (Gibb et al., 2023). Psyllium has benefits such as treatment of diarrhea, constipation, inflammatory bowel disease, hypercholesterolemia, and diabetes. Study showed that psyllium consumption decreases blood glucose levels after one dose consumption (Belorio & Gómez, 2021; Darooghegi Mofrad et al., 2020; Gibb et al., 2015; Singh, 2007). Consumption of both soluble and insoluble fiber leads to decrease in blood pressure (Lupton & Turner, 2003). Consuming psyllium reduces the risk of developing many cardiovascular diseases, including high cholesterol, hypertriglyceridemia, hyperglycemia, and hypertension (Cicero et al., 2007). In the treatment of cardiometabolic diseases like hyperlipidemia, diabetes mellitus, and its complications such as hypertension, hyperuricemia, and obesity, as well as in the use of food systems, psyllium is used (Chen et al., 2022). In high‐normal blood pressure subjects who consume a lot of salt, regular consumption of dietary fiber made from psyllium husk lowers SBP (Yoshinuma et al., 2019). The addition of fiber to one's diet may yield favorable outcomes on vascular health and blood pressure. A study was conducted to investigate the impacts of 12 weeks of psyllium fiber consumption on blood pressure and vascular function in obese and overweight persons. However, the results of this study did not indicate any significant improvements in either of these markers (Pal et al., 2012). The consumption of psyllium supplementation led to a notable reduction in SBP, while there was no significant decrease observed in DBP (Clark et al., 2020). But in another study, 6 months of administration of psyllium fiber showed a significant decrease in both SBP and DBP among overweight individuals with hypertension (Cicero et al., 2007).

Psyllium consumption may affect BP; the ingestion of nutrients within the lumen is linked with a reduction in postprandial glucose levels and an improvement in systemic insulin resistance. This process heightens insulin sensitivity and regulates the glycemic response. Moreover, it stimulates the production of nitric oxide, a vasodilator, which promotes blood vessel dilation (Jane et al., 2019). The intake of viscous fiber is a major pathophysiological mechanism for the development of endothelial dysfunction and hypertension. This is due to the increase in food viscosity, which slows down nutrient absorption in the lumen and causes compensatory insulinemia. There are various associated mechanisms such as increased renal sodium reabsorption, activation of the sympathetic nervous system, alteration in transmembrane ion transport, and hypertrophy of resistance vessels. These mechanisms are partially mediated by activation of the mitogen‐activated protein kinase pathway (Clark et al., 2020). A decrease in insulin resistance has also been put forth by the means of action psyllium which lowers blood pressure (Gibb et al., 2023). The contribution of both soluble and insoluble fibers in the reduction of insulin resistance and insulin levels could aid in the treatment or prevention of hypertension in individuals with or without diabetes (King et al., 2005; Qi et al., 2005).

### 4.1 Strengths and limitations

This study has several strengths that should be acknowledged: (a) this was, to our knowledge, the meta‐analysis with more studies than the other studies that evaluate the effect of psyllium on SBP and DBP; (b) we performed a detailed sensitivity analysis; (c) predefined subgroup analyses were conducted to ascertain the origins of heterogeneity between studies; (d) we conducted a dose–response meta‐analysis; and (e) last study (Clark et al., 2020) considered 11 trials in 2018 but we considered 14 studies until 2023. However, concomitant to the noted strengths, some limitations should be considered in the interpretation of our findings. For instance: (a) some of the included studies did not account for the dietary intake, which is known to potentially affect SBP and DBP; (b) we restricted the number of the included studies; (c) the age range of included participants was wide; (d) we had unidentified heterogeneity in several of the results; (e) the majority of the included studies were very small and used various psyllium types and doses during various intervention times; (f) different health status existed among the included subjects, and some significant confounders were left uncontrolled; and (g) only 802 individuals—a relatively small number—are present in the literature used in this meta‐analysis.

## 5 CONCLUSION

This study conducted a systematic review and meta‐analysis to explore the impact of psyllium on systolic blood pressure (SBP) and diastolic blood pressure (DBP) due to the inconsistent findings in existing research. Accordingly, we found that psyllium could significantly decrease SBP levels, but non‐significant decrease in SBP, as compared to placebo. Therefore, we advocate that psyllium should be considered as a potential treatment option, if clinically appropriate.

## AUTHOR CONTRIBUTIONS

**Zamzam Paknahad:** Supervision (equal); writing – review and editing (equal). **Zeinab Gholami:** Conceptualization (equal); data curation (equal); formal analysis (equal); investigation (equal); methodology (equal); project administration (equal); resources (equal); software (equal); writing – original draft (equal); writing – review and editing (equal).

## FUNDING INFORMATION

The present study has been financially supported by a grant from Student Research Committee, Isfahan University of Medical Sciences (Code: IR.MUI.RESEARCH.REC.1402.048, Grant number: 140222).

## CONFLICT OF INTEREST STATEMENT

The authors affirm that they do not possess any identifiable financial interests or personal affiliations that could have potentially influenced the research presented in this manuscript.

## ETHICS STATEMENT

It has received prior approval from the Medical Ethics Committee of Isfahan University of Medical Sciences. (Code: IR.MUI.RESEARCH.REC.1402.048, Grant number: 140222). This study is registered in PROSPERO, under code CRD42023417492.

## Supporting information


Data S1

## Data Availability

The datasets generated and/or analyzed during the current study are not publicly available due to some restrictions applied by the ethics committee, but are available from the corresponding author on reasonable request.

## References

[fsn33863-bib-0001] Anderson, J. W. , Zettwoch, N. , Feldman, T. , Tietyen‐Clark, J. , Oeltgen, P. , & Bishop, C. W. (1988). Cholesterol‐lowering effects of psyllium hydrophilic mucilloid for hypercholesterolemic men. Archives of Internal Medicine, 148(2), 292–296. 3277558

[fsn33863-bib-0002] Bell, L. P. , Hectorne, K. , Reynolds, H. , Balm, T. K. , & Hunninghake, D. B. (1989). Cholesterol‐lowering effects of psyllium hydrophilic mucilloid: Adjunct therapy to a prudent diet for patients with mild to moderate hypercholesterolemia. Journal of the American Medical Association, 261(23), 3419–3423. 2724486 10.1001/jama.261.23.3419

[fsn33863-bib-0003] Belorio, M. , & Gómez, M. (2021). Psyllium: A useful functional ingredient in food systems. Critical Reviews in Food Science and Nutrition, 62(2), 527–538. 10.1080/10408398.2020.1822276 32951436

[fsn33863-bib-0004] Borenstein M , Hedges L , Higgins J , Rothstein H . Comprehensive meta‐analysis, version 2 biostat. Biostat. 2005.

[fsn33863-bib-0005] Chen, C. , Shang, C. , Xin, L. , Xiang, M. , Wang, Y. , Shen, Z. , Jiao, L. , Ding, F. , & Cui, X. (2022). Beneficial effects of psyllium on the prevention and treatment of cardiometabolic diseases. Food & Function, 13(14), 7473–7486. 35781477 10.1039/d2fo00560c

[fsn33863-bib-0006] Cicero, A. F. , Derosa, G. , Bove, M. , Imola, F. , Borghi, C. , & Gaddi, A. V. (2010). Psyllium improves dyslipidaemia, hyperglycaemia and hypertension, while guar gum reduces body weight more rapidly in patients affected by metabolic syndrome following an AHA Step 2 diet. Mediterranean Journal of Nutrition and Metabolism, 3(1), 47–54.

[fsn33863-bib-0007] Cicero, A. F. , Derosa, G. , Manca, M. , Bove, M. , Borghi, C. , & Gaddi, A. V. (2007). Different effect of psyllium and guar dietary supplementation on blood pressure control in hypertensive overweight patients: A six‐month, randomized clinical trial. Clinical and Experimental Hypertension, 29(6), 383–394. 17729055 10.1080/10641960701578378

[fsn33863-bib-0008] Cicero, A. F. , Fogacci, F. , Stoian, A. P. , Vrablik, M. , Al Rasadi, K. , Banach, M. , Toth, P. P. , & Rizzo, M. (2021). Nutraceuticals in the management of dyslipidemia: Which, when, and for whom? Could nutraceuticals help low‐risk individuals with non‐optimal lipid levels? Current Atherosclerosis Reports, 23, 1–14. 10.1007/s11883-021-00955-y PMC8332568 34345932

[fsn33863-bib-0009] Clark, C. C. , Salek, M. , Aghabagheri, E. , & Jafarnejad, S. (2020). The effect of psyllium supplementation on blood pressure: A systematic review and meta‐analysis of randomized controlled trials. The Korean Journal of Internal Medicine, 35(6), 1385–1399. 32066221 10.3904/kjim.2019.049 PMC7652639

[fsn33863-bib-0010] Darooghegi Mofrad, M. , Mozaffari, H. , Mousavi, S. M. , Sheikhi, A. , & Milajerdi, A. (2020). The effects of psyllium supplementation on body weight, body mass index and waist circumference in adults: A systematic review and dose‐response meta‐analysis of randomized controlled trials. Critical Reviews in Food Science and Nutrition, 60(5), 859–872. 30880409 10.1080/10408398.2018.1553140

[fsn33863-bib-0011] DerSimonian, R. , & Laird, N. (1986). Meta‐analysis in clinical trials. Controlled Clinical Trials, 7(3), 177–188. 3802833 10.1016/0197-2456(86)90046-2

[fsn33863-bib-0012] Gibb, R. D. , McRorie, J. W., Jr. , Russell, D. A. , Hasselblad, V. , & D'Alessio, D. A. (2015). Psyllium fiber improves glycemic control proportional to loss of glycemic control: A meta‐analysis of data in euglycemic subjects, patients at risk of type 2 diabetes mellitus, and patients being treated for type 2 diabetes mellitus. The American Journal of Clinical Nutrition, 102(6), 1604–1614. 26561625 10.3945/ajcn.115.106989

[fsn33863-bib-0013] Gibb, R. D. , Sloan, K. J. , & McRorie, J. W., Jr. (2023). Psyllium is a natural nonfermented gel‐forming fiber that is effective for weight loss: A comprehensive review and meta‐analysis. Journal of the American Association of Nurse Practitioners, 35(8), 468–476. 37163454 10.1097/JXX.0000000000000882 PMC10389520

[fsn33863-bib-0014] Higgins, J. P. , Altman, D. G. , Gøtzsche, P. C. , Jüni, P. , Moher, D. , Oxman, A. D. , Savovic, J. , Schulz, K. F. , Weeks, L. , Sterne, J. A. , & Cochrane Bias Methods Group; Cochrane Statistical Methods Group . (2011). The Cochrane Collaboration's tool for assessing risk of bias in randomised trials. BMJ, 343, d5928. 22008217 10.1136/bmj.d5928 PMC3196245

[fsn33863-bib-0015] Higgins, J. P. , Thompson, S. G. , Deeks, J. J. , & Altman, D. G. (2003). Measuring inconsistency in meta‐analyses. BMJ, 327(7414), 557–560. 12958120 10.1136/bmj.327.7414.557 PMC192859

[fsn33863-bib-0016] Jane, M. , McKay, J. , & Pal, S. (2019). Effects of daily consumption of psyllium, oat bran and polyGlycopleX on obesity‐related disease risk factors: A critical review. Nutrition, 57, 84–91. 30153584 10.1016/j.nut.2018.05.036

[fsn33863-bib-0017] Jenkins, D. , Wolever, T. , Vidgen, E. , Kendall, C. , Ransom, T. , Mehling, C. , Mueller, S. , Cunnane, S. C. , O'Connell, N. C. , Setchell, K. D. , Lau, H. , Teitel, J. M. , Garvey, M. B. , Fulgoni, V., 3rd , Connelly, P. W. , Patten, R. , & Corey, P. N. (1997). Effect of psyllium in hypercholesterolemia at two monounsaturated fatty acid intakes. The American Journal of Clinical Nutrition, 65(5), 1524–1533. 9129487 10.1093/ajcn/65.5.1524

[fsn33863-bib-0018] Jovanovski, E. , Yashpal, S. , Komishon, A. , Zurbau, A. , Blanco Mejia, S. , Ho, H. V. T. , Li, D. , Sievenpiper, J. , Duvnjak, L. , & Vuksan, V. (2018). Effect of psyllium (*Plantago ovata*) fiber on LDL cholesterol and alternative lipid targets, non‐HDL cholesterol and apolipoprotein B: A systematic review and meta‐analysis of randomized controlled trials. The American Journal of Clinical Nutrition, 108(5), 922–932. 30239559 10.1093/ajcn/nqy115

[fsn33863-bib-0019] Kearney, P. M. , Whelton, M. , Reynolds, K. , Whelton, P. K. , & He, J. (2004). Worldwide prevalence of hypertension: A systematic review. Journal of Hypertension, 22(1), 11–19. 15106785 10.1097/00004872-200401000-00003

[fsn33863-bib-0020] Khan, K. , Jovanovski, E. , Ho, H. , Marques, A. , Zurbau, A. , Mejia, S. B. , Sievenpiper, J. L. , & Vuksan, V. (2018). The effect of viscous soluble fiber on blood pressure: A systematic review and meta‐analysis of randomized controlled trials. Nutrition, Metabolism and Cardiovascular Diseases, 28(1), 3–13. 10.1016/j.numecd.2017.09.007 29153856

[fsn33863-bib-0021] King, D. E. , Mainous, A. G., III , Egan, B. M. , Woolson, R. F. , & Geesey, M. E. (2005). Fiber and C‐reactive protein in diabetes, hypertension, and obesity. Diabetes Care, 28(6), 1487–1489. 15920074 10.2337/diacare.28.6.1487

[fsn33863-bib-0022] Lupton, J. R. , & Turner, N. D. (2003). Dietary fiber and coronary disease: Does the evidence support an association? Current Atherosclerosis Reports, 5(6), 500–505. 14525684 10.1007/s11883-003-0041-y

[fsn33863-bib-0023] Mandoki, J. J. , Casa‐Tirao, B. , Molina‐Guarneros, J. A. , Jiménez‐Orozco, F. A. , García‐Mondragón, M. J. , & Maldonado‐Espinoza, A. (2013). Pulsatile diastolic increase and systolic decrease in arterial blood pressure: Their mechanism of production and physiological role. Progress in Biophysics and Molecular Biology, 112(3), 55–57. 23727290 10.1016/j.pbiomolbio.2013.05.002

[fsn33863-bib-0024] McRorie, J., Jr. , Gibb, R. , Sloan, K. , & McKeown, N. (2021). Psyllium: The gel‐forming nonfermented isolated fiber that delivers multiple fiber‐related health benefits. Nutrition Today, 56(4), 169–182.

[fsn33863-bib-0025] Naghdi Badi, H. , Dastpak, A. , & Ziai, S. (2004). A review of psyllium plant. Journal of Medicinal Plants, 3(9), 1–14.

[fsn33863-bib-0026] Neal, G. , & Balm, T. (1990). Synergistic effects of psyllium in the dietary treatment of hypercholesterolemia. Southern Medical Journal, 83(10), 1131–1137. 2218650 10.1097/00007611-199010000-00005

[fsn33863-bib-0027] Olson, B. H. , Anderson, S. M. , Becker, M. P. , Anderson, J. W. , Hunninghake, D. B. , Jenkins, D. J. , LaRosa, J. C. , Rippe, J. M. , Roberts, D. C. , Stoy, D. B. , Summerbell, C. D. , Truswell, A. S. , Wolever, T. M. , Morris, D. H. , & Fulgoni, V. L., 3rd . (1997). Psyllium‐enriched cereals lower blood total cholesterol and LDL cholesterol, but not HDL cholesterol, in hypercholesterolemic adults: Results of a meta‐analysis. The Journal of Nutrition, 127(10), 1973–1980. 9311953 10.1093/jn/127.10.1973

[fsn33863-bib-0028] Pal, S. , Khossousi, A. , Binns, C. , Dhaliwal, S. , & Radavelli‐Bagatini, S. (2012). The effects of 12‐week psyllium fibre supplementation or healthy diet on blood pressure and arterial stiffness in overweight and obese individuals. British Journal of Nutrition, 107(5), 725–734. 21787454 10.1017/S0007114511003497

[fsn33863-bib-0029] Parums, D. V. (2021). Review articles, systematic reviews, meta‐analysis, and the updated preferred reporting items for systematic reviews and meta‐analyses (PRISMA) 2020 guidelines. Medical Science Monitor: International Medical Journal of Experimental and Clinical Research, 27, e934475‐1. 34421116 10.12659/MSM.934475 PMC8394590

[fsn33863-bib-0030] Qi, L. , Rimm, E. , Liu, S. , Rifai, N. , & Hu, F. B. (2005). Dietary glycemic index, glycemic load, cereal fiber, and plasma adiponectin concentration in diabetic men. Diabetes Care, 28(5), 1022–1028. 15855561 10.2337/diacare.28.5.1022

[fsn33863-bib-0031] Ricklefs‐Johnson, K. , Johnston, C. , & Sweazea, K. (2017). Ground flaxseed increased nitric oxide levels in adults with type 2 diabetes: A randomized comparative effectiveness study of supplemental flaxseed and psyllium fiber. Obesity Medicine, 5, 16–24.

[fsn33863-bib-0032] Sartore, G. , Reitano, R. , Barison, A. , Magnanini, P. , Cosma, C. , Burlina, S. , Manzato, E. , Fedele, D. , & Lapolla, A. (2009). The effects of psyllium on lipoproteins in type II diabetic patients. European Journal of Clinical Nutrition, 63(10), 1269–1271. 19623196 10.1038/ejcn.2009.60

[fsn33863-bib-0033] Singh, B. (2007). Psyllium as therapeutic and drug delivery agent. International Journal of Pharmaceutics, 334(1–2), 1–14. 17329047 10.1016/j.ijpharm.2007.01.028

[fsn33863-bib-0034] Skrypnik, D. , Skrypnik, K. , Pelczyńska, M. , Sobieska, M. , Tinkov, A. A. , Suliburska, J. , & Bogdański, P. (2021). The effect of Plantago major supplementation on leptin and VEGF‐A serum levels, endothelial dysfunction and angiogenesis in obese women–a randomized trial. Food & Function, 12(4), 1708–1718. 33502416 10.1039/d0fo01878c

[fsn33863-bib-0035] Solà, R. , Bruckert, E. , Valls, R.‐M. , Narejos, S. , Luque, X. , Castro‐Cabezas, M. , Doménech, G. , Torres, F. , Heras, M. , Farrés, X. , Vaquer, J. V. , Martínez, J. M. , Almaraz, M. C. , & Anguera, A. (2010). Soluble fiber (*Plantago ovata* husk) reduces plasma low‐density lipoprotein (LDL) cholesterol, triglycerides, insulin, oxidized LDL and systolic blood pressure in hypercholesterolaemic patients: A randomized trial. Atherosclerosis, 211(2), 630–637. 20413122 10.1016/j.atherosclerosis.2010.03.010

[fsn33863-bib-0036] Staessen, J. A. , Wang, J. , Bianchi, G. , & Birkenhäger, W. H. (2003). Essential hypertension. The Lancet, 361(9369), 1629–1641. 10.1016/S0140-6736(03)13302-8 12747893

[fsn33863-bib-0037] Tabrizi, L. , Nasiri, M. M. , & Kouchaki, A. R. (2005). Investigations on the cardinal temperatures for germination of *Plantago ovata* and Plantago psyllium.

[fsn33863-bib-0038] Wei, Z. , Wang, H. , Chen, X. , Wang, B. , Rong, Z. , Su, B. , & Chen, H. Z. (2009). Time‐and dose‐dependent effect of psyllium on serum lipids in mild‐to‐moderate hypercholesterolemia: A meta‐analysis of controlled clinical trials. European Journal of Clinical Nutrition, 63(7), 821–827. 18985059 10.1038/ejcn.2008.49

[fsn33863-bib-0039] Wen, O. P. , Zakaria, N. S. , Kamarudin, K. S. , & Yusof, H. M. (2022). Effects of short‐term psyllium husk and selected mixed herbal supplementation on health indicators in healthy male subjects. Journal of Applied Pharmaceutical Science, 12(2), 126–132.

[fsn33863-bib-0040] Wolever, T. , Jenkins, D. , Mueller, S. , Boctor, D. L. , Ransom, T. , Patten, R. , Chao, E. S. , & McMillan, K. (1994). Fulgoni III V Method of administration influences the serum cholesterol–lowering effect of psyllium. The American Journal of Clinical Nutrition, 59(5), 1055–1059. 8172091 10.1093/ajcn/59.5.1055

[fsn33863-bib-0041] Yoshinuma, H. , Inoike, N. , Tanabe, S. , Ebihara, S. , & Nakamura, F. (2019). Effects of intake of psyllium husk on blood pressure in subjects with high‐normal blood pressure ‐randomized, double‐blind, placebo‐controlled parallel‐group study. Japanese Pharmacology and Therapeutics, 47(9), 1519–1527.

