# Supplementary material for: The effect of psyllium consumption on blood pressure: Systematic review and dose–response meta‐analysis of randomized controlled trials
Source: Food Sci Nutr. 2024 Aug 29;12(10):7075–87. doi: 10.1002/fsn3.3863 (PMC11521634; doi:10.1002/fsn3.3863)
Supplement: Supplementary file 1 — Data S1 [file FSN3-12-7075-s001.docx]

### (a)

### (b)

### Supplementary figure 1: sensitivity analysis of psyllium on SBP (a) and DBP (b)

(a)

(b)

### Supplementary figure 2: dosage subgroup analyses for the effects of psyllium on SBP (a) and DBP (b)

**(a)**

**(b)**

**Supplementary figure 3:** duration subgroup analyses for the effects of psyllium on SBP (a) and DBP(b)

### (a)

###

### (b)

###

**Supplementary figure 4:** Random-effects meta-regression plots of the association between mean changes in SBP (a) and DBP (b) and intervention duration

### (a)

###

### (b)

###

**Supplementary figure 5:** Publication bias of psyllium on SBP (a) and DBP (b)
